# Surface Water Transitions 1984–2022: A Global Dataset at Annual Resolution

**DOI:** 10.1038/s41597-025-06013-5

**Published:** 2025-10-31

**Authors:** Gustavo Willy Nagel, Stephen E. Darby, Julian Leyland

**Affiliations:** School of Geography and Environmental Sciences, University of Southampton, Southampton, SO17 1BJ UK

**Keywords:** Hydrology, Environmental impact, Physical oceanography

## Abstract

Recent advances in satellite technology and cloud computing have enabled global-scale monitoring of long-term surface water changes. The dynamic nature of surface water, driven by seasonal fluctuations and climatic events, presents challenges for accurately interpreting these dynamics. Here, we introduce the first global dataset that identifies the timing, at annual resolution, of surface water advance or recession from 1984 to 2022. Our approach focuses on identifying persistent changes in surface water features by filtering out seasonal or shorter-term fluctuations. Using a novel algorithm, we mapped the timing of surface water transitions globally, including rivers, lakes, reservoirs, flooded agriculture, and coastal regions. In the dataset each 30 m × 30 m pixel records whether water advance or recession occurred and specifies the year of transition. This dataset enables users to visualize the location, type, and magnitude of changes, while its focus on timing provides new insights into the drivers of water dynamics. Designed for accessibility, the dataset supports scientific research as well as NGOs, policymakers, and water managers in addressing surface water-related challenges.

## Background & Summary

Surface water plays a fundamental role in shaping our planet’s landscapes and supporting human development^1–6^. The dynamic interplay between wet and dry conditions has driven profound changes throughout Earth’s history, sculpting landforms, influencing ecosystem dynamics, and impacting human civilizations. Changes in surface water extent can be attributed variously to human activities such as urban expansion and the construction of dams and levees, as well as to natural processes such as river meandering^2,7–15^. Climate change also influences the distribution of surface water through its impacts on precipitation variability, temperature changes and melting of ice^16–18^. Establishing the location and magnitude of changes in surface water extent is, therefore, important in a variety of contexts^6,19^. However, understanding the *timing* of transitions of surface water state (these transitions being defined here as a persistent and significant shift in the state of surface water, such as when a previously wet area becomes permanently dry, or a dry area becomes consistently inundated) is critical in causal inference.

To address the challenge of identifying changes in the location and extent of surface water bodies, in recent years researchers have turned to satellite imagery and advances in cloud computing to enable its detection at the global scale. Examples of such prior studies include mapping the presence or absence of surface water, assessing its temporal occurrence, monitoring reservoir dynamics, and identifying areas of long-term surface water loss and gain^12,19–21^. Pekel, *et al*.^20^, Donchyts, *et al*.^19^ and Pickens, *et al*.^21^ have each made significant contributions to this field by mapping global surface water extent and net changes in extent over periods of multiple decades using Landsat imagery. However, whilst these previous approaches have helped in identifying the global distribution of changing surface water extent across many years, they do not specifically seek to pinpoint the timing of such changes, potentially limiting their utility for evaluating causation.

In this study we present the first global analysis of the timing of surface water transitions (at annual resolution) using Landsat satellites images from 1984 to 2022 within the Google Earth Engine (GEE) cloud computing environment. Herein we define such temporal transitions as occurring either when a pixel was dry, and subsequently becomes persistently flooded or with seasonal water presence (inundated during the flooding period of the region), or when a pixel was wet, and subsequently becomes persistently dry or experiences only scarce flooding events (which occur less frequently than inundation periods). Our new dataset is publicly available on GEE and is also readily accessible through a GEE app that aids visual interpretation (https://ee-gustavoonagel.projects.earthengine.app/view/water-change-time-detection). The novelty and utility of our research lies in its emphasis on understanding the timing of surface water transitions at fine (annual) temporal resolution, which thereby enables these transitions to be linked more clearly to their driving factors, potentially allowing a clearer assessment of the implications of such transition timings for landscape evolution and human development.

**Fig. 1:**
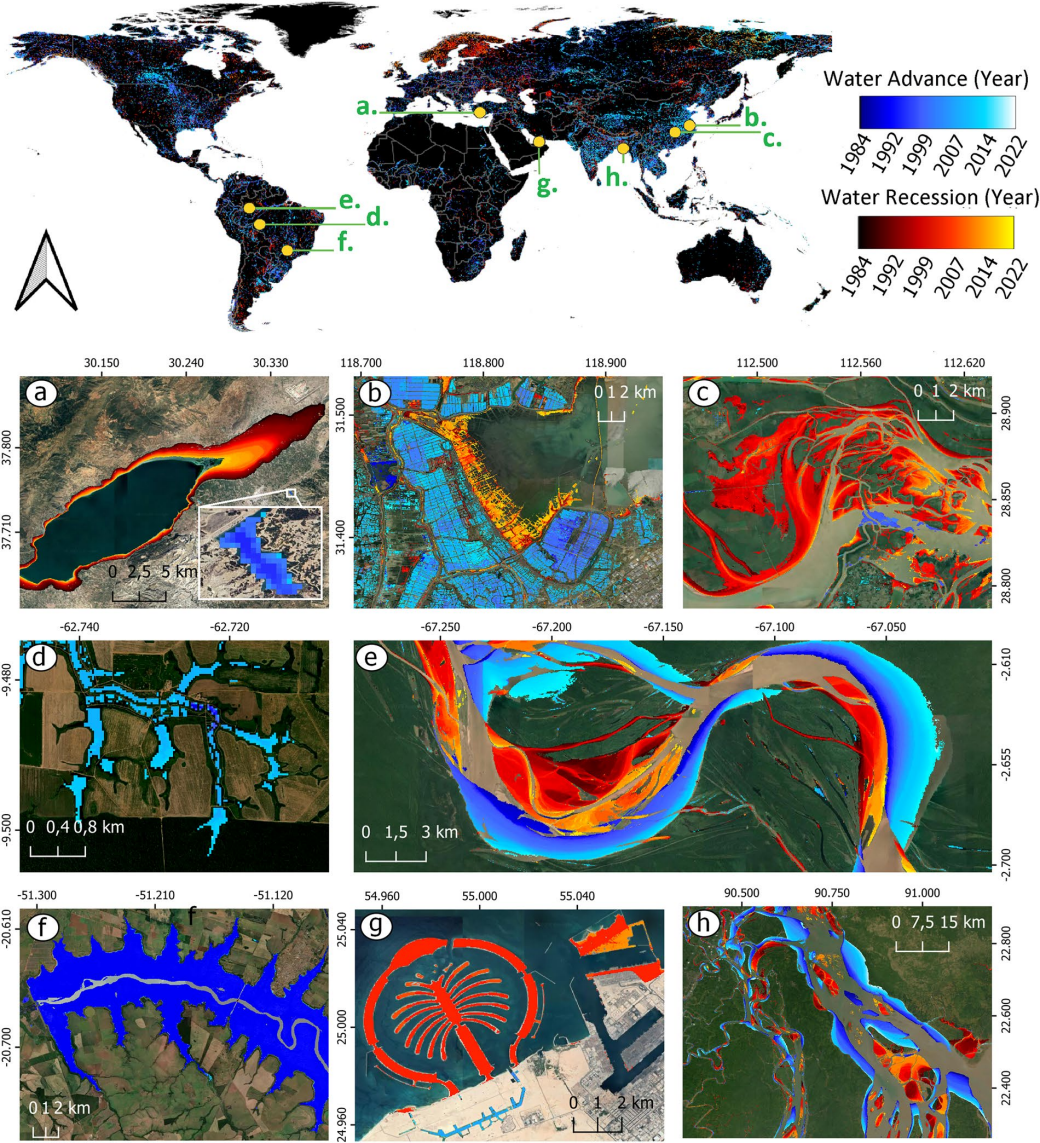
A global perspective on the timing of surface water transitions (water advance or recession events). Examples of natural and engineered changes show: the drying of Lake Burdur (an important wetland area and one of the deepest lakes in Türkiye) (**a**), flood-based agricultural expansion in China (**b**), a floodplain in China (**c**), the expansion of small reservoirs for fishing and cattle rearing in deforested areas of Amazonia (**d**), the meandering of the Amazon River (**e**), the establishment of the lake behind the Três Irmãos hydroelectric dam in Brazil (**f**), the construction of artificial islands in Dubai (**g**), and the delta of the Ganges River in Bangladesh (**h**).

Our surface water transition dataset provides a comprehensive representation of surface water temporal variability, capturing both the timings, locations, extents, and intricate spatial patterns of change influenced by diverse natural and anthropogenic processes (Fig. 1). For example, in regions like China (Fig. 1b) and Dubai (Fig. 1g), distinctive patterns^22^ shaped by human land reclamation and food production activities stand in contrast to the natural patterns of change observed on rivers and floodplains (Fig. 1c,e,h). Additionally, our dataset reveals that human interventions tend to drive very rapid surface water transitions, as is the case with the creation of the Três Irmãos Reservoir in Brazil (Fig. 1f) that occurred abruptly in 1991, the construction of the Palm Islands in Dubai (Fig. 1g), which experienced its peak of land reclamation in 2005, or the fish lakes constructed in 2015 following Amazonian deforestation (Fig. 1d). Furthermore, different spatial scales of change are also captured, as exemplified in Fig. 1a, which shows a lake of approximately 70.2 km² that experienced drought and a nearby lake of only 0.08 km² that was created in 1994.

### Improved spatial visualization of water temporal dynamics

Water is a highly dynamic resource with intricate spatial and temporal variations, presenting significant challenges for effective monitoring and visualization. Various statistical approaches and classification techniques can be employed to represent temporal changes in raster datasets, such as mapping water occurrence and identifying regions where water has expanded or receded over time^19–21^. Our dataset, on the other hand, is the first to explicitly record the year of surface water transition, distinguishing between periods of water advance and water recession. Furthermore, our dataset was designed to be intuitive and easy to interpret, allowing users to quickly identify periods of significant surface water advance or recession, different patterns of change, as well as the direction of movement, improving predictability and trend analysis. Due to its accessibility, the dataset is not only valuable for academic research but also serves as a practical resource for governments, water management agencies, NGOs, and the general public, enabling informed decision-making and promoting sustainable water management.

## Methods

The unique combination of its high spatial resolution (30 m), long duration of archive (spanning from 1984 for Landsat 5 through to 2023 for Landsat 9), and a revisit time of 16 days positions the Landsat program as a highly suitable tool for undertaking a comprehensive analysis of global inland and coastal surface water extents. These characteristics explain the widespread use of Landsat time series (merging images from the Landsat 5 to Landsat 9) in a range of prior studies^6,12,15,19,20,23,24^. Here our methodological approach employed automated analysis of Landsat imagery to detect pixels demonstrating a persistent change in surface water presence over time, as characterized by a consistent trend of either increasing or decreasing extent. Subsequently, we pinpointed the specific year in which each pixel transitioned from dry-dominated to water-dominated states (or *vice versa*). While we aimed to minimize the impact of short-lived (sub-annual) fluctuations in water extent in order to identify the timing of ‘permanent’ surface water change, it’s important to note that our ability to capture long-term fluctuations is constrained by historical data limitations. In this study, we define permanent change as change that involves an enduring shift in water frequency throughout the 38-year analysis period. We acknowledge that this definition does not imply the immutability of water conditions in these locations; future changes in surface water advance or recession may, of course, still occur. The consideration of permanent change within the context of our analysis relates only to observed shifts within the studied (1984–2022) timeframe.

To accurately detect the timing (year) of surface water change, we developed two new algorithms utilizing Landsat Time Series from 1984 to 2022 and the cloud computing platform Google Earth Engine (GEE). The first algorithm, the Water Change Detection (WCD) algorithm, estimates areas of surface water transition by leveraging the Modified Normalized Difference Water Index (mNDWI)^25–34^, while the second algorithm, here termed the Water Time Track Detection (WTTD) algorithm, identifies the year of change through the use of the Green_Red Normalized Difference Water Index (grNDWI) proposed herein.

### Water index

Water indexes use a combination of satellite bands to identify water features. These water feature identification methods have been used extensively to detect surface water resources in different environments^25,26,35^. Although they have different equations, water indexes normally rely on the use of bands that are highly absorbed by water (such as infrared) and bands less absorbed by water (visible spectrum)^25^. To produce binary water maps, a threshold is applied to separate land (non-water) from water pixels. To detect the time that water permanently advanced and/or receded we applied different water indexes, regional and local optimum thresholds, and methods to avoid high-frequency fluctuations in water extent using Google Earth Engine (GEE). We divided our analysis into two parts. First, an algorithm identifies areas where the water advanced and/or receded (Water Change Detection Algorithm – WCD), while the second algorithm estimates the year that the change occurred (Water Time Track Detection – WTTD).

Landsat’s use of different infrared bands allows for a variety of water indexes to be employed, including the popular Normalized Difference Water Index (NDWI)^36^, which uses a normalized difference between the green and near infrared (NIR) bands (see Eq. 1), and the Modified Normalized Difference Water Index (mNDWI)^25^, which uses the mid-infrared (SWIR) instead of the NIR band (Eq. 2). However, each index has its own advantages and limitations. As a result, we tested the mNDWI, the NDWI and our proposed Green/Red Normalized Difference Index (grNDWI – Eq. 3), which uses a normalization between the green-red mean and the NIR, across different environments and land classes. In the literature, a good water index is usually characterized as one that is capable of reliably differentiating water from land^37,38^. However, in addition to this, to be able to track changes *through time* a good water index also needs to have a smooth and consistent transition from land to water and vice versa, as explained below.

1 $${NDWI}=\frac{{Green}-{NIR}}{{Green}+{NIR}}$$

2 $${mNDWI}=\frac{{Green}-{SWIR}1}{{Green}+{SWIR}1}$$

3 $${grNDWI}=\frac{\left({Green}+{Red}\right)/2-{NIR}}{\left({Green}+{Red}\right)/2+{NIR}}$$

Green = Landsat 5 (Band 2 = 0.52 μm–0.60 μm)/Landsat 8 and 9 (Band 3 = 0.53 μm–0.59 μm)

Red = Landsat 5 (Band 3 = 0.63 μm–0.69 μm)/Landsat 8 and 9 (Band 4 = 0.64 μm–0.67 μm)

Near Infrared (NIR) = Landsat 5 (Band 4 = 0.76 μm–0.90 μm)/Landsat 8 and 9 (Band 5 = 0.85 μm–0.88 μm)

SWIR1 = Landsat 5 (Band 7 = 2.08 μm–2.35 μm)/Landsat 8 and 9 (Band 7 = 2.11 μm–2.29 μm)

**Fig. 2:**
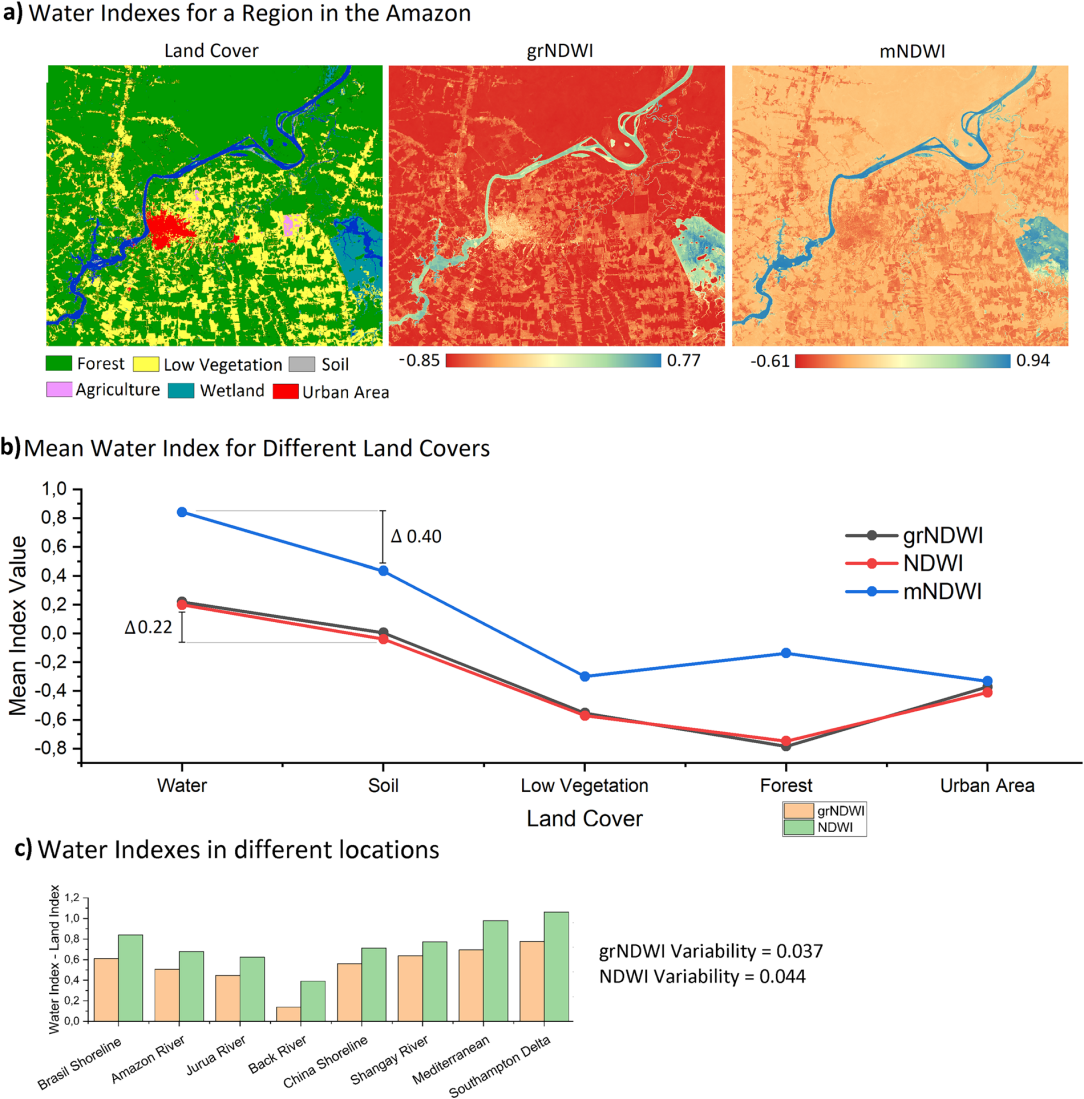
Comparison of the mNDWI, NDWI, and grNDWI indices for a region within the Amazon Basin (capital of Rondônia State, Brazil) (**a**). The mean value of each water index was calculated for specific land cover types, and then compared to simulate a temporal transition from exposed soil to low vegetation, forest, and urban areas, respectively (**b**). Additionally, a comparative analysis between the grNDWI and mNDWI indices was conducted across different locations (**c**).

Figure 2 provides an analysis of the derived mNDWI, NDWI, and grNDWI water indices in the Amazon region, specifically in Rondônia State, Brazil. This region was selected because it is characterised by a range of land cover types, including dense forest, low vegetation (grassland), urban areas, and complex turbidity dynamics, that potentially affect the different water indices. Figure 2 showcases the mean water index values obtained across these different land covers, simulating the temporal transition from exposed soil to low vegetation, forest, and urban areas (Fig. 2). The mNDWI demonstrates a higher water-land index difference (0.4 from water to soil) compared to either of the grNDWI and NDWI indices, in principle making it more suitable both for water detection and for identifying pixel-level changes in water presence over time. However, the mNDWI exhibits higher values for forest compared to low vegetation, which deviates from the expected index reduction as land cover transitions towards drier conditions. In contrast, the grNDWI and NDWI indices exhibit consistent decreases from water to forest, following an almost linear relationship (R² of 0.96), and then increasing again in urban areas. Consequently, for natural environments, the grNDWI and NDWI offer a more consistent transition from water to land, making them better candidates for identifying the year of change.

However, while the mNDWI uses the SWIR band, and so is less affected by water quality parameters, both grNDWI and NDWI use the NIR band, which is sensitive to sediment content in the water^39^ and thus can be affected by a river that changes its turbidity over time. Therefore, the proposed grNDWI that we employ here uses the red band (Eq. 3) to counterbalance this effect, creating an index that is less affected by turbidity variability. We tested this hypothesis by comparing a water and a land index pixel (Difference = water pixel – land pixel) from grNDWI and NDWI to identify which one had the lower variability between the different locations (Fig. 2c). Indexes with lower variability between locations with different water turbidity concentrations will perform better in identifying the surface water year of change. We selected pixels from the Amazon, Black and Jurua Rivers, Brazil China’s and the Mediterranean shorelines, the Yangtze Delta, and the Solent, UK, and confirmed that the grNDWI index has 15.7% less variability than the NDWI. As a result, we used the mNDWI to identify areas of water advance and/or recession (within the WCD algorithm) and the grNDWI to estimate the year of change (within the WTTD algorithm).

### Water threshold

**Fig. 3:**
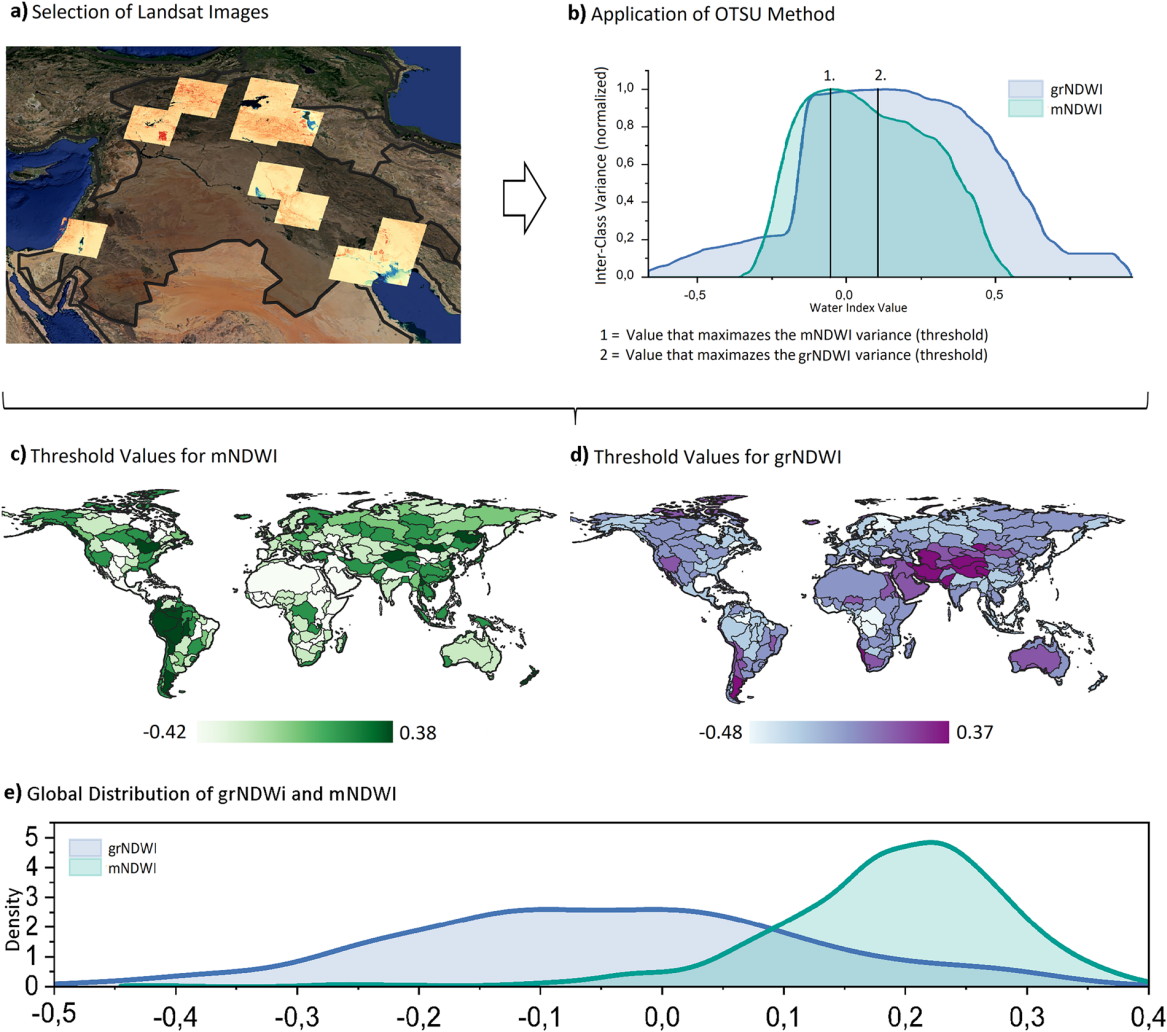
OTSU methodology (**a** and **b**), the threshold results for different regions of the World (**c** and **d**), and the global threshold value distribution (**e**).

To derive meaningful information from a water index, it is essential to apply a threshold to identify water pixels accurately. To account for the spatial variability in water index values caused by differences in water quality parameters and surrounding environments, we implemented a zone-based approach. This involved dividing the world into distinct zones based on global watersheds^40^ and along the coast, where watersheds and regions with similar true colour characteristics, which are related to the land cover, were visually merged using a satellite database provided by Google^41^ (a total of 244 such regions were created). We adopted a watershed-based approach to ensure consistent thresholding within individual water bodies and to avoid assigning different thresholds to the same hydrological system. The merging of watersheds allowed us to adjust the watershed boundaries to better reflect coherent environmental regions, including forests, deserts, and savannas. Within each zone, we aimed to determine an optimum threshold that effectively separates water from land using both the grNDWI and mNDWI indices. To obtain representative data for each zone, we employed an automated process to select the least cloudy Landsat images in areas with potential water presence. If a Landsat image spanned multiple watersheds, the method clipped the image to retain only the portions falling within each individual watershed boundary. Additionally, we applied cloud and snow masks to exclude areas affected by cloud cover and snow accumulation. Figure 3 presents an example of the least cloudy images selected for a region in the Middle East, deliberately excluding waterless desert areas. With this approach, we created regional thresholds that remain relatively stable across space and within the same hydrological system, minimizing artificial variability in the classification results. However, since pixel-level thresholds can vary abruptly due to changes in water quality and surrounding land cover, we developed a complementary method (referred to as the Pixel Threshold) which is explained in the following section.

Subsequently, we applied the OTSU Method^42,43^ to automatically identify the threshold value. This method determines the threshold by maximizing the variability between two classes, in this case, water and land (Fig. 3b), while minimizing the differences within each class. To ensure a balanced representation of water and land for applying the Otsu thresholding method, we utilized water occurrence data from Pekel, *et al*.^20^ to identify areas with consistently high water presence and areas with low or no water occurrence. From these, we generated random samples for each image, half drawn from high-frequency water zones and half from low or zero-frequency zones, representing land. This sampling strategy was applied individually to each image. The original Otsu method computes the threshold using all available pixels in an image, which is computationally intensive and impractical for large-scale applications. By restricting input to a balanced and representative subset of pixels, our approach maintains the essential land–water contrast required for effective thresholding while significantly improving computational efficiency. The mNDWI threshold values obtained through the OTSU Method were utilized for the WCD, while the grNDWI threshold values were applied for the WTTD algorithms. As explained before, mNDWI is less affected by water quality parameters, so the range of global mNDWI is lower than the grNDWI (the inclusion of the red band improves this effect but does not eliminate the water quality influence) (Fig. 3d). By employing zone-specific thresholds, we account for the spatial variations in water index values, ensuring accurate detection and estimation of permanent water changes in different regions.

###  Algorithms for Water Change and Temporal Tracking Detection

**Fig. 4:**
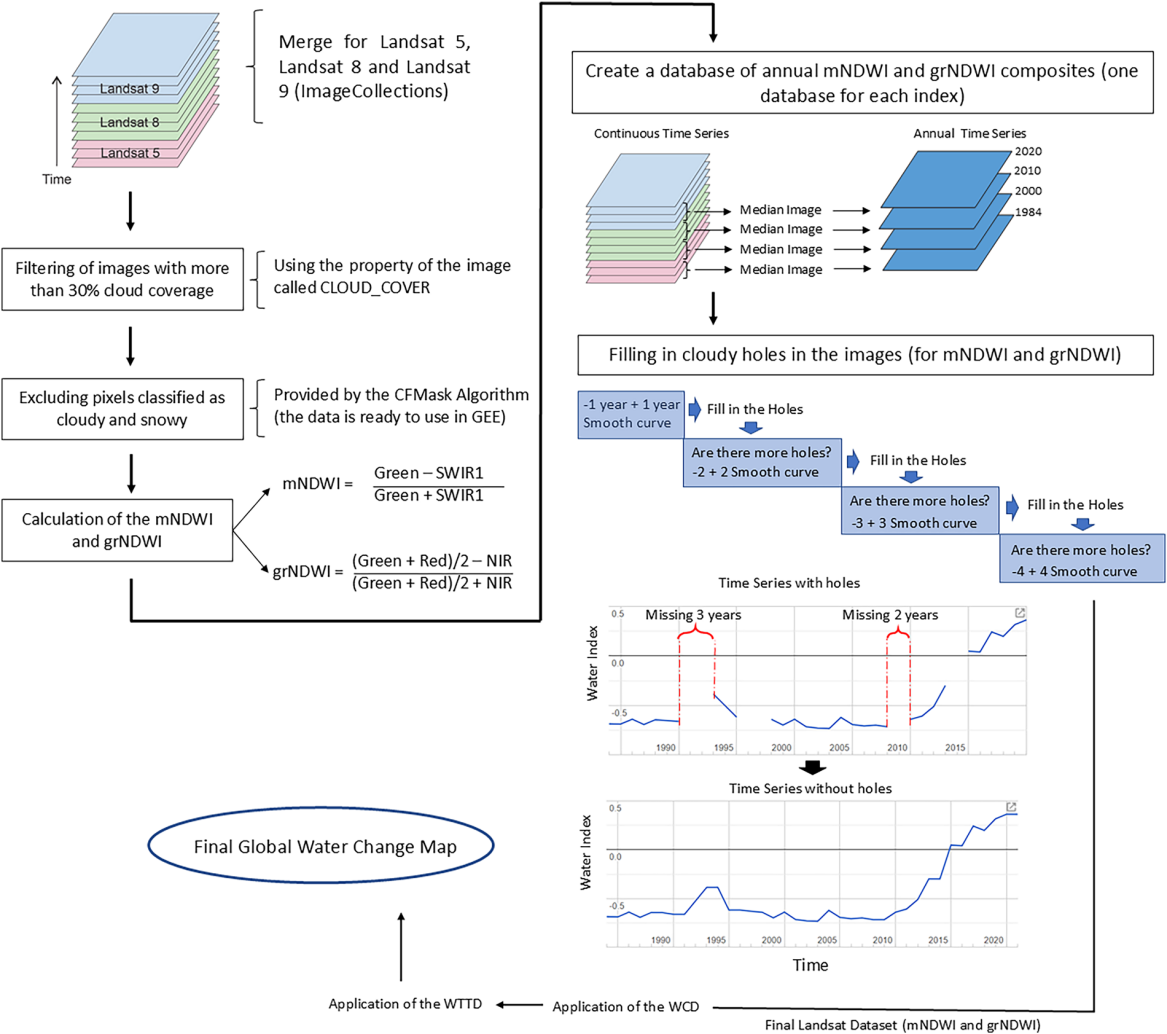
Flowchart of the pre-processing steps for WCD and WTTD.

To ensure the suitability of the Landsat image database for the application of the WCD and WTTD algorithms, a series of pre-processing steps were implemented. These steps were designed to enhance the quality and consistency of the data (see Fig. 4). The following procedures were executed:

i) The Landsat 5, 8, and 9 images were merged to create a comprehensive ImageCollection within the Google Earth Engine (GEE) platform, totalling 3,982,821 images.
ii) To mitigate the influence of cloud cover on the analysis, images with cloud coverage exceeding 30% were filtered out from the dataset (which reduced the available data to 55% of the original dataset = 2,190,743 images).
iii) A cloud and snow mask, derived from the CFMask product, was applied to the remaining images to identify and remove cloud/snow pixels.
iv) The mNDWI and grNDWI indexes were calculated for each image.
v) Two separate annual composites (one based on mNDWI and another on grNDWI) were generated by calculating the median pixel values for each year. These composites represent the water dynamics for each respective year.

**Fig. 5:**
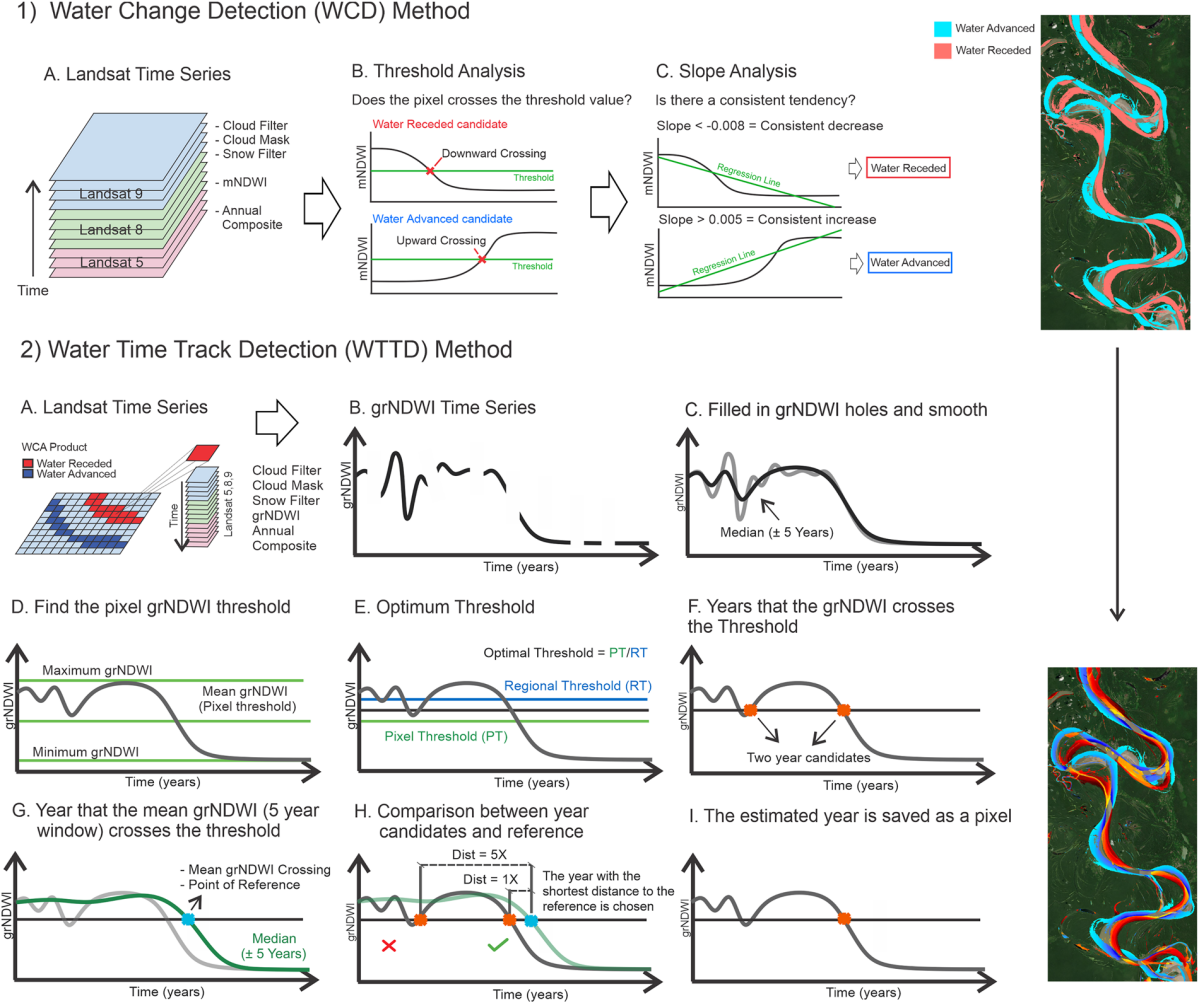
WCD and the WTTD algorithm flowcharts.

The new pre-processed Landsat collection was used to delineate areas of changing surface water extent using the Water Change Detection (WCD) algorithm on annual Modified Normalized Difference Water Index (mNDWI). Through a pixel-wise analysis, areas of potential change were identified based on the crossing of a pre-determined mNDWI threshold, indicating either water advance (upward crossing) or water recession (downward crossing) (Fig. 5b). Recognizing the dynamic nature of surface water, where regions may experience recurrent episodes of wetting or drying, we employed a further analysis of each pixel’s mNDWI trend, the trend being determined from Ordinary Least Squares Regression, using the GEE function ee.Reducer.linearFit(), as computed using annual mNDWI over time. Pixels that exhibited both an upward crossing of the threshold and a regression line slope exceeding 0.005 mNDWI/year were considered areas of water advance. Conversely, pixels that displayed both a downward crossing of the threshold and a trend of less than −0.008 mNDWI/year were classified as regions of water recession (Fig. 5c).

To establish the slope thresholds, we gathered 10,000 points from regions worldwide where we already knew surface water had either advanced or receded. Following a method developed by Nagel, *et al*.^15^, these slope values were then sorted, and the 5^th^ percentile values were extracted for water-recession (−0.008 ∆mNDWI/year) and water-advance (0.005 ∆mNDWI/year) pixels. Consequently, pixels with a slope below −0.008 ∆mNDWI/year were classified as having a negative trend, while pixels with a slope above 0.005 ∆mNDWI/year were classified as having a positive trend.

To determine the timing (here we resolve timing at annual resolution) of water advance or recession, we employed the WTTD algorithm (Fig. 5) in areas identified by the WCD algorithm as experiencing water advance or recession. The WTTD algorithm was specifically designed to filter out short-lived events, such as seasonal flooding and droughts. To perform the WTTD, we first smoothed the grNDWI using a ±5 years window. The average difference between the original and filtered series, based on 1,200 randomly selected locations, was −0.015. Furthermore, the standard deviation of the differences (0.139) was substantially smaller than that of both the original (0.330) and filtered series (0.303), suggesting the filter smooths the data without distorting the original pattern. Then an additional pre-processing technique was executed to address the issue of missing data in certain years, in which the algorithm interpolated a new value based on the preceding and succeeding year (Fig. 5e – details are provided in Fig. 4). If the data gap extended beyond one year, the algorithm extends the time interval by considering images from two years prior and two years after the missing year. This process is applied iteratively, with the algorithm expanding the time interval until a year with available data was found.

Then, with the continuous and spatially coherent Landsat image database, we extracted the maximum and minimum grNDWI values to calculate the mean grNDWI, which served as the pixel-specific water-land threshold (Fig. 5.2f). This pixel threshold represents a locally optimized water/land threshold, acknowledging that a pixel transitioning from forest to turbid water would have a different threshold than a pixel transitioning from sand to clear water, even if they were located in close proximity. We note that, by relying on the entire time series, the pixel threshold method is susceptible to potential outliers, such as unfiltered clouds and atmospheric constituents, which can affect the accuracy of the results. To address this, we computed the mean between the individual pixel threshold and a regional threshold (calculated using the OTSU method, see Fig. 3), resulting in the creation of the Optimal Threshold that incorporates both local and regional influences (Fig. 5g). While the Optimal Threshold may introduce local noisiness in regions exhibiting high variability in land cover, particularly due to its influence on the maximum value assigned to land (for instance, forests generating a different grNDWI than sandy areas), it plays a crucial role in the global and regional scales that are the focus of this work. The Optimal Threshold allows for the adjustment of the threshold based on distinct water quality environments and land covers and is, therefore, essential to prevent the generation of inaccurate results that may arise if such variations are not taken into account.

The newly derived Optimal Threshold serves as the basis for determining the transition year of water advance or recession. For any individual pixel to be defined as experiencing water advance, it must cross the Optimal Threshold in an upward direction, while water recession pixels must cross it in a downward direction, as depicted in Fig. 5h. Considering the influence of short-lived wetting events, an individual pixel may cross the Optimal Threshold multiple times, resulting in several candidate transition years (Fig. 5h). To select the actual year of transition used in the analysis, we calculated the mean grNDWI using a 5-year moving window and determined the year at which it crossed the Optimal Threshold, establishing a reference year (Fig. 5i). By comparing the candidate years with the reference year, we selected the candidate with the smallest difference (Fig. 5j). In the provided example, we encountered two candidate years and chose the more recent one (Fig. 5k). The transition year is then stored as part of the pixel dataset that covers the entire Earth.

### Data quality

**Fig. 6:**
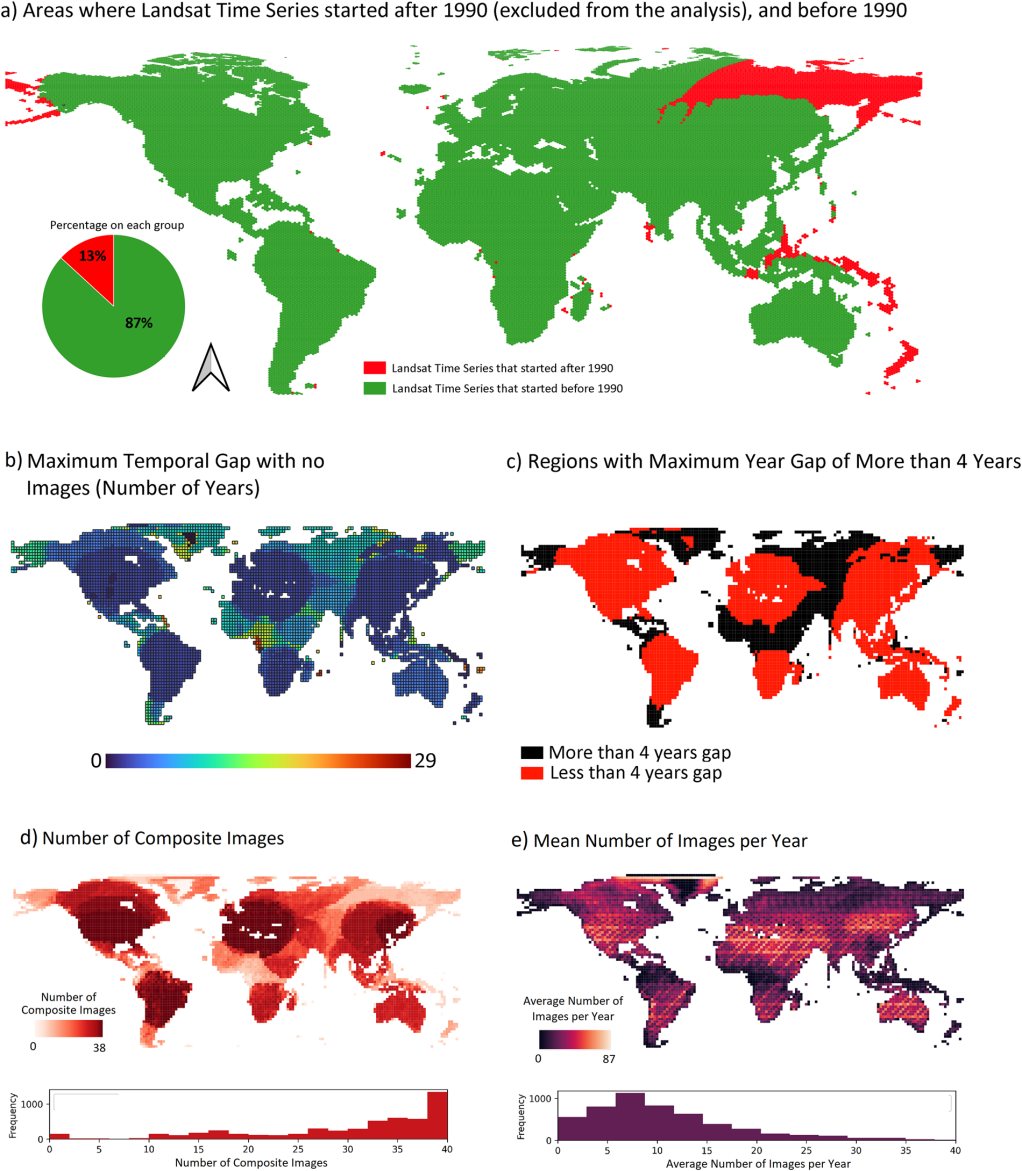
Areas where Landsat Time Series started after 1990 (excluded from the analysis) and before 1990 (**a**), the maximum temporal gap without images in the Time Series (**b**), the regions with less than 4 years of maximum temporal gap (**c**), the number of yearly composite images (**d**) and the Mean Number of Images per year (**e**).

To ensure the accuracy of the WCD algorithm in identifying trends of water advance and/or recession, it was necessary to have a sufficient number of years of Landsat time series data. Therefore, we excluded regions where the Landsat time series began after 1990, which accounted for only 13% of the global surface (Fig. 6a). This exclusion was necessary to maintain the reliability and consistency of the analysis by focusing on areas with longer temporal coverage of Landsat data. Furthermore, most (82%—each composite representing one year) (Fig. 6d) regions have more than 30 composite images, with an average of around 7 images per year used to create each composite (Fig. 6e). Data gaps are a major problem for the accuracy of the water detection. However, the majority of Earth’s surface has data gaps of three years or less (51.1% of regions) (Fig. 6d). Furthermore, these gaps only introduce imprecision in the detection of transition timing when they coincide with the transition process, such as the precise moment when a pixel is shifting to wetter or drier conditions, or vice versa. Based on an analysis of 1,200 randomly selected points we found that only 1% of water advance and recession years were detected in periods with no grNDWI information.

**Fig. 7:**
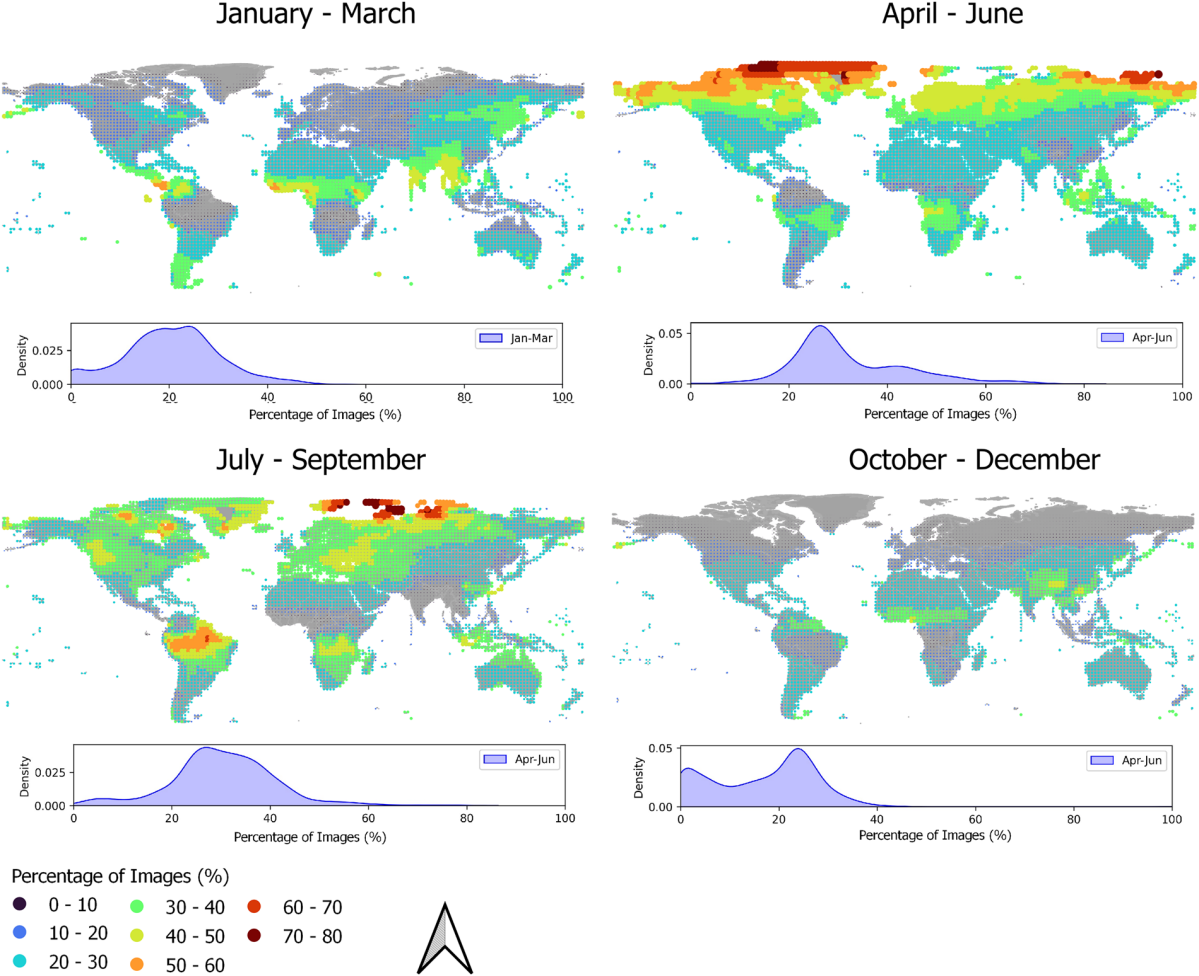
Percentage of Landsat images available for different seasons (January-March, April-June, July-September, October-December). A distribution of values (percentage of images) related to each season is presented below the map. Since the year was divided into four different periods, a value of 25% of available images would represent a perfectly even distribution of images throughout the year.

We analyzed the temporal distribution of available data across different months (Fig. 7) and assessed the corresponding patterns of availability. The year was divided into four calendar-based seasons, which are not necessarily aligned with climatic seasons due to differences between the northern and southern hemispheres. Ideally, an even distribution would result in 25% of the data in each season. Our analysis shows that, while there are regional patterns, such as a concentration of images in the Amazon between July and September and at higher latitudes between April and June, the overall data distribution is well-balanced. Most areas have data availability ranging between 20% and 25% for each of the four seasons (see the Percentage of Image distribution for each period in Fig. 7), indicating a good overall distribution throughout the year.

## Data Records

**Fig. 8:**
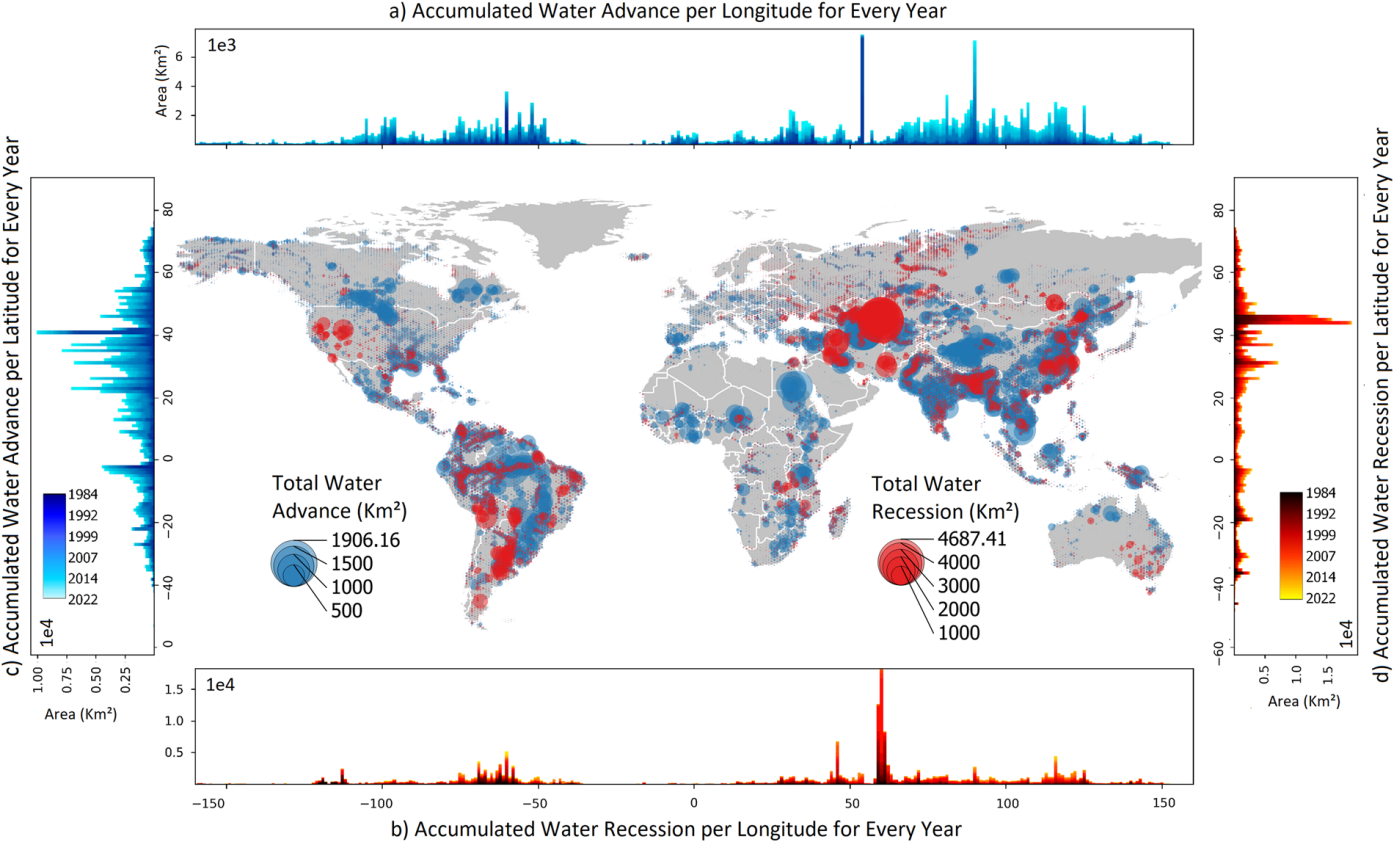
Global distribution of total water advance and recession, plotted at a regional scale of 1,010 km^2^. Point size indicates the magnitude of water advance or recession. (**a**) Accumulated water advance by longitude for each year. (**b**) Accumulated water recession by longitude for each year. (**c**) Total water advance by latitude for each year. (**d**) Total water recession by latitude for every year.

The Water Transition Dataset is hosted on Figshare^44^ and features a global TIF file that maps the timing of water transitions, with areas of water advance and recession stored in two distinct bands. To ensure transparency and reproducibility, the dataset also provides a GeoJSON file containing key metadata, such as regions with Landsat time series post-1990, the number of composite images, the average number of images per year, and the percentage of Landsat data available across different seasons (illustrated in Figs. 6 and 7). Additionally, the JavaScript code used to generate the water transition imagery is included in a TXT file. As the dataset was processed in small regional segments and later merged, the code example specifically focuses on the Ganges Delta. The Water Transition Dataset is based on pixel-level identification of water change. Figure 8 presents these results using a coarser (1,010 km²) global grid to explore regional patterns (with larger points indicating greater water change in that area). This regional-scale gridded representation is for visualization purposes only and does not modify the underlying pixel-level data. Note that, at the regional-scale, both water advance and recession can appear within the same (1,010 km²) grid cell, reflecting the coexistence of different local trends in nearby water bodies.

## Technical Validation

**Fig. 9:**
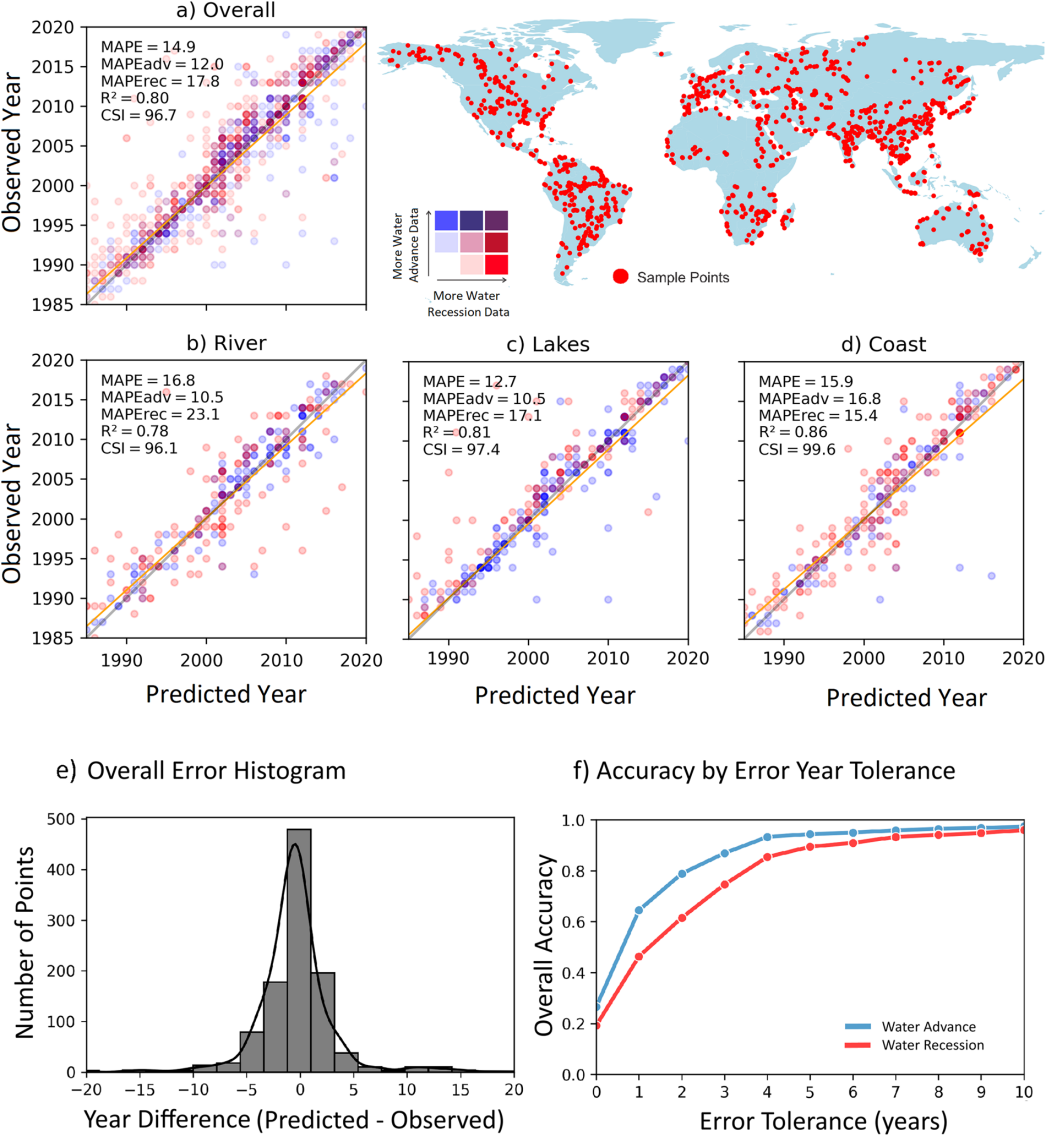
Validation of the Water Transition Dataset for the entire globe (**a**) and for rivers (**b**), lakes (**c**), and coasts (**d**). The overall distribution of errors is shown in (**e**), and the overall accuracy across different error tolerances is shown in (**f**).

We conducted a validation of the water detection algorithm by collecting 1,111 geographically diverse data points across the globe: this sample size is, according to Slovin’s formula^45^, sufficient to generate a 3% margin of error^46,47^. To facilitate the validation process, we developed a user-friendly Google Earth Engine (GEE) App that produced a random distribution of points and displayed a Landsat Time Series with the NIR, Red, and Blue band combination to highlight distinctions between surface water and land. Through the careful visualization of approximately 42,218 Landsat scenes (1,111 × 38 years), we determined the year of transition in relation to water advance and recession and compared that year with the year of change as estimated using the WTTD algorithm described above. The determination of the transition year was performed by identifying the year that water advanced at each point and remained there, and the year that the water receded and remained dry. This validation procedure encompassed the full range of water bodies evaluated in the analysis (i.e., rivers, lakes and coastal environments). Key statistical metrics were derived to evaluate the performance of the WTTD algorithm: the Mean Absolute Percentage Error for combined water advance and recession points (MAPE), MAPE for water advance points (MAPEadv), MAPE for recession points (MAPErec), the Coefficient of Determination (R²), the Critical Success Index (CSI), and the Overall Accuracy, thereby establishing a comprehensive assessment framework (see Fig. 9).

Our analysis yielded a Mean Absolute Percentage Error (MAPE) of 14.9%, indicating a high level of reliability. The coefficient of determination (R²) was 0.80, demonstrating a strong correlation between the predicted and observed data points, while the overall CSI was 96.7% when considering all water bodies in a single regression model (Fig. 9a). These statistics indicate the robustness of the WTTD algorithm, considering the intricate and dynamic nature of global water surfaces. Notably, the accuracy of the WTTD algorithm is seen to vary across different Water Environment Classes, with lakes and coasts exhibiting the best results (Fig. 9c,d), characterized by MAPE values of 12.7% and 15.9%, CSI values of 96% and 98.9%, and high R² values of 0.81 and 0.86, for water recession and water advance respectively. Rivers (MAPE: 15.5%, CSI: 95.5%, and R²: 0.78) also demonstrated good accuracy (Fig. 9b). Moreover, it is important to highlight that regions of water advance generally exhibited better estimations compared to water receding regions. The predicted year matched the observed year exactly in 26.5% of the Water Advance samples and 19.2% of the Water Recession samples (Fig. 9f). When the error tolerance is increased to ±1 year, the overall accuracy rises to 64.5% for Water Advance and 46.3% for Water Recession. Accuracy continues to improve with increasing tolerance, reaching 94.4% and 89.4%, respectively, at a ±5-year tolerance. Notably, even at a more conservative tolerance of ±3 years, the method demonstrates strong overall accuracy, with 87.0% for Water Advance and 74.7% for Water Recession, as illustrated in Fig. 9f.

These variations in accuracy can be attributed to the inherent complexity of different water features and the extent to which water colour and water extent change over time, which directly impact the performances of both the WCD and WTTD algorithms. Coastal regions, for instance, are influenced by a combination of factors such as ocean currents, wave energy, tides, and coastal morphometric characteristics^48,49^. However, these influences tend to persist over longer durations, and it is less likely for an eroding coast to transition into a sediment-filled one within the period of our analysis. Additionally, coastal areas generally exhibit lower complexity in terms of their water colour when compared to inland water bodies, conditions that increased the accuracy of the water change algorithms. On the other hand, rivers are subject to interannual rainfall variations, seasonality effects, and water turbidity fluctuations, which introduce uncertainties in the analysis. Lake environments generally exhibit higher overall accuracy, primarily attributed to the rapid global construction of new dams^50^. Our water change algorithms can reliably detect the year when a reservoir is filled, as it undergoes a transition from a dry to a persistently wetted state. However, lakes that are highly dynamic and sensitive to rainfall conditions tend to have lower accuracy.

Nonetheless, certain characteristics are common to all water features. In all water features, pixels representing water advance tend to have higher accuracy compared to pixels indicating water recession. This is primarily because inundation or erosion processes tend to occur more rapidly, making it easier to identify the transition year. In the case of meandering rivers, for instance, erosion typically occurs on the deeper outer bank, which experiences lower seasonality. Conversely, sedimentation gradually accumulates over time in the shallower inner bank, which is more influenced by seasonal fluctuations, a more complex condition that reduces the accuracy of temporal tracking of surface water.

## Data Availability

The JavaScript code used to generate the water transition imagery is hosted on Figshare and is provided in a TXT file. To run the code, copy and paste its contents into the Google Earth Engine (GEE) code editor. The region of interest can be specified by setting the latitude and longitude values in the variable “coordinates”; the code is structured to adapt to the selected area without requiring additional modifications. The provided example focuses on the Ganges Delta, as the dataset was processed in smaller regional segments and later merged.
